# Nur77 deficiency in mice accelerates tumor invasion and metastasis by facilitating TNFα secretion and lowering CSF-1R expression

**DOI:** 10.1371/journal.pone.0171347

**Published:** 2017-02-07

**Authors:** Xiu-Ming Li, Jing-Ru Wang, Tong Shen, Shang-Shang Gao, Xiao-Shun He, Jiang-Nan Li, Tian-Yu Yang, Shen Zhang, Wen-Juan Gan, Jian-Ming Li, Hua Wu

**Affiliations:** 1 Pathology Center and Department of Pathology, Soochow University, Suzhou, China; 2 Department of Pathology, The First Affiliated Hospital of Soochow University, Suzhou, China; 3 Department of Pathology, Sun Yat-sen Memorial Hospital, Sun Yat-sen University, Guangzhou, China; University of South Alabama Mitchell Cancer Institute, UNITED STATES

## Abstract

Nur77, an orphan member of the nuclear receptor superfamily, plays critical roles in inflammation and immunity. However, the role of Nur77 in tumor microenvironment remains elusive. Results showed that deletion of Nur77 strikingly enhanced tumor metastasis compared to WT mice. Additionally, compared to the conditioned media derived from Nur77^+/+^ peritoneal macrophages (CM1), the conditioned media derived from Nur77^-/-^ peritoneal macrophages (CM2) significantly promoted the EMT of cancer cells, and greatly enhanced the migratory and invasive abilities of cancer cells. Moreover, studies using TNF-α blocking antibody demonstrated that pro-inflammatory cytokine TNF-α was indispensable in supporting CM2-induced EMT to drive cancer cells migration and invasion. Furthermore, we found that Nur77 promoted the expression of CSF-1R, a novel downstream target gene of Nur77, and subsequently enhanced the migration of inflammatory cells. Notably, infiltration of inflammatory cells in the tumors of Nur77^-/-^ mice was markedly abrogated compared to Nur77^+/+^ mice. Collectively, these results revealed that host Nur77 expression was pivotal in antitumor immune response, and in inhibiting tumor metastasis.

## Introduction

Tumor microenvironment (TME) comprises an abundance of inflammatory and immune cells, and inflammatory mediators, all of which collaborate in the development and progression of cancer [1]. Evidence increasingly suggests that aberrant inflammation mediated by inflammatory and immune cells, such as macrophages, dendritic cells, and lymphocytes, is associated with an increased risk of human diseases including cancer [2, 3]. Inflammatory and immune cells in TME can lead to an antitumor response or pro-tumor support of growth, survival, invasion, and metastasis through secretion of several cytokines, including tumor necrosis factor-alpha (TNF-α) [1, 4–7]. Increased TNF-α levels have been observed in patients with ovarian and breast cancer [8, 9], and is important in promoting tumorigenesis and metastasis. These studies indicate that inflammatory and immune cells in the TME have a vital role in cancer development and progression. However, the underlying regulatory mechanism of inflammatory and immune cells in tumor development and progression remains unclear.

Nur77 (also known as TR3, NGFIB, or NR4A1), an orphan member of the nuclear receptor superfamily, serves as an important regulator in cancer and inflammatory diseases [10–13]. Elevated Nur77 expression is often observed in cancer including colorectal cancer, bladder cancer and pancreatic cancer [14–17], indicating that Nur77 acts as a tumor promotor to drive cancer cell growth and metastasis. For example, overexpression of Nur77 in colorectal cancer promotes cancer cell invasion and metastasis by regulating MMP-9-dependent E-cadherin reduction [15]. In pancreatic and colorectal cancers, knockdown of Nur77 significantly inhibits cell growth [14, 17]. Conversely, many studies have suggested that Nur77 plays a protective role in inflammatory diseases. For example, mice lacking Nur77 expression accelerated atherosclerosis *via* regulation of the polarization of macrophages [18]. We recently also reported that Nur77 deficiency in elderly mice leads to systemic inflammation [19], and lack of Nur77 increases the susceptibility of mice to LPS-induced sepsis and DSS-induced inflammatory bowel disease (IBD) by modulating Toll-like receptor (TLR) signaling [20, 21]. Mounting evidence has shown that chronic, persistent inflammation contributes to all stages of tumor development, so the question arises as to whether Nur77 deficiency in inflammatory and immune cells regulates tumor development.

To establish the role of Nur77 in TME, tumor metastatic spreading was investigated in an *in vivo* metastasis model in wild-type (Nur77^+/+^) mice and Nur77^-/-^ mice. Our results showed that expression of host Nur77 was a critical factor in antitumor metastasis because in its absence metastatic spreading was greatly accelerated. Furthermore, our study revealed two potential key mechanisms by which the absence of Nur77 expression in inflammatory cells drove cancer cells invasion and metastasis. On one hand, lack of Nur77 in macrophages promoted inflammatory cytokine TNF-α production, which stimulated cancer cells to undergo EMT and endowed them invasive properties. On the other hand, Nur77-deficient macrophages had low tumor-infiltrating migratory ability due to downregulation of CSF-1R expression, a novel target gene of Nur77. Altogether these results unveil a novel function of Nur77 in regulating tumor invasion and metastasis.

## Materials and methods

### Antibody and regents

Anti-Vimentin, anti-E-cadherin and anti-Myc tag antibodies were purchased from Santa Cruz Biotechnology (Santa Cruz, CA, USA). Anti-β-actin antibodies were purchased from Sigma-Aldrich (St. Louis, MO, USA). Anti-CSF-1R antibodies were purchased from Boster (Wuhan, China). Lipofectamine 2000 and TRIzol reagents were purchased from Invitrogen (Carlsbad, CA, USA) and WesternBright ECL reagents were purchased from Advansta (Menlo Park, CA, USA).

### Cell culture and transfection

RAW264.7, THP-1, CT-26 and B16 cells were purchased from the Cell Bank of the Chinese Academy of Sciences (Shanghai, China) and maintained in RPMI 1640 media containing 10% fetal bovine serum (FBS) in a humidified atmosphere containing 5% CO_2_ at 37°C. RAW264.7 and THP-1 cells were transfected with vector or myc-Nur77 plasmid using Lipofectamine 2000. All cell lines were passaged in the laboratory for fewer than 2 months after resuscitation and were used at the third through seventh passage in culture for this study.

### Mice

Nur77^+/+^ and Nur77^−/−^ mice were purchased from the Jackson Laboratory. All mice were maintained in a specific pathogen-free environment with a 12-hour/12-hour light-dark cycle at the Laboratory Animal Center in Soochow University (China), and were provided with normal laboratory pellet diet and water. The mice feed was sterilized by irradiation with 60Co-γ ray. All of the experiments were designed in strict accordance with the guidelines of the Animal Care and Use Committee of Soochow University. Animals were monitored daily by the animal facility caretakers and weekly by a staff veterinarian. Mice that became unexpectedly ill or exhibited signs of distress were removed from the study, anesthetized then sacrificed by cervical dislocation.

*In vivo* experiments were performed with Nur77^+/+^ and Nur77^−/−^ mice in accordance with the guidelines of the Animal Care and Use Committee of Soochow. A total of 0.5 × 10^6^ B16 cells were intravenously injected into WT (Nur77^+/+^) mice and Nur77^-/-^ mice (C57, SPF grade, 11 months, male). Six weeks after tumor cell administration, the mice of each group were sacrificed by cervical dislocation. Tumor nodes on lung or liver were counted and showed by H&E staining.

### Isolation of peritoneal macrophages and collection of conditioned media

Peritoneal macrophages were isolated as described recently [21]. Briefly, mice were injected intraperitoneally (i.p.) with 2 ml of 4% thioglycolate (Sigma) for 3 d, then we washed the peritoneal cavity with ice-cold DMEM and the cells in the peritoneal exudates were isolated. Collected cells were incubated in a humidified atmosphere containing 5% CO_2_ at 37°C for 4 h, and adherent cells were harvested as peritoneal macrophages. The peritoneal macrophages were continually cultured with media for 3 day, and then conditioned media (CM) was harvested.

### Western blot and immunofluorescence staining

Western blot was performed as recently described [22, 23]. Briefly, the protein from cell lysates were separated by 8%-10% SDS-PAGE gel, and then the divided proteins were transferred to a PVDF membrane (Millipore). Protein expression was detected using primary and secondary antibodies, and visualized using enhanced chemiluminescence reagents and autoradiography. Cells for immunofluorescence staining were grown and stained as previously described [24]. The images were taken with a Nikon ECLIPSE Ni scope with color camera and were processed by NIS-Elements D 4.10.00 software.

### RNA extraction and qPCR analysis

The total RNAs were extracted using TRIzol LS (Invitrogen). The reverse transcriptional reaction was performed using RevertAid^™^ First Strand cDNA Synthesis Kits (Fermentas). qPCR was performed using Power SYBR^®^ Green PCR Master Mix (TaKaRa, Japan). Normalization was performed with *β-actin*. Primers with the following sequences were used: *CSF-1R* (Human), forward 5′-TGCTGCTCCTGCTGCTATTG-3′ and reverse 5′-TCAGCATCTTCACAGCCAC -C-3′; *CSF-1R* (Mouse), forward 5′-CCACCATCCACTTGTATGTCAAAGAT-3′ and reverse 5′-CTCAACCACTGTCACCTCCTGT-3′; *β-actin* (Human) forward 5′-CACCAACTGGGACGACATG-3′ and reverse 5′- GCACAGCCTGGATAGCAA -C-3′; *β-actin* (Mouse) forward 5′-TGGAATCCTGTGGCATCCATGAAAC-3′ and reverse 5′- TAAAACGCAGCTCAGTAACAGTCCG-3′.

### CSF-1R promotor constructs and luciferase assays

The sequence of CSF-1R promotor was obtained from human genomic DNA isolated from HEK293T cells and inserted into KpnⅠand XhoⅠ sites of pGL3-basic luciferase reporter vector. The activity of luciferase reporter was determined in HEK293T cells 36 h after transfection of the indicated plasmids. Each transfection also included β-galactosidase (β-gal) for normalization of luciferase activity.

### Chromatin immunoprecipitation (ChIP)

ChIP was performed as previously described [14]. Briefly, cells were cross-linked with 1% formaldehyde (Sigma) for 10 min at room temperature and then quenched with 125 μM glycine and incubated for 5 min at room temperature. Cross-linked chromatin was sheared to an average length of 500 bp through sonication and subjected to immunoprecipitation with antibodies against anti-Myc tag antibody or with nonimmune IgG as controls overnight at 4°C, followed by incubation with protein A beads (GE Healthcare) for 2 h. After washing and elution, the protein-DNAcomplex was reversed by adding NaCl and proteinase K for 2 hours at 65°C. Immunoprecipitated DNA was purified by using QIAquick spin columns (Qiagen) and analyzed by qPCR. All ChIP qPCRs were performed at least three times and representative results are shown. Primers with the following sequences were used: forward 5′-TAACGGTCTGGCTGCTCTTG-3′ and reverse 5′- AGCTAAGTTTGGGGCCCTTC-3′.

### Transwell migration and Matrigel invasion assays

For transwell migration assay, 5 × 10^4^ CT-26 cells in 500 μl serum-free DMEM medium were added to the apical chamber, and the basal chamber was filled with 500 μl conditioned media. After 24 h, the cells located at the underside of the filter were fixed with 100% methanol for 5 min and then stained with Wright-Giemsa at room temperature. For Matrigel invasion assay, BD BioCoat Matrigel Invasion Chambers (Catalog No. 354480) were used for the invasion assay according to the instructions of the manufacturer. Images of the cells were taken by inverted microscope (magnification, 200×). To quantify the migratory and invasive cells microscopically, cells were counted in five random fields.

### Statistical analysis

Each assay was performed in three independent experiments. Data were presented as mean ± s.d. Statistical significance was analyzed using Student's *t* test (unpaired, two-tailed) and *p* < 0.05 was considered statistically significant.

## Results

### Accelerated tumor metastasis in Nur77-KO mice

**Fig 1:**
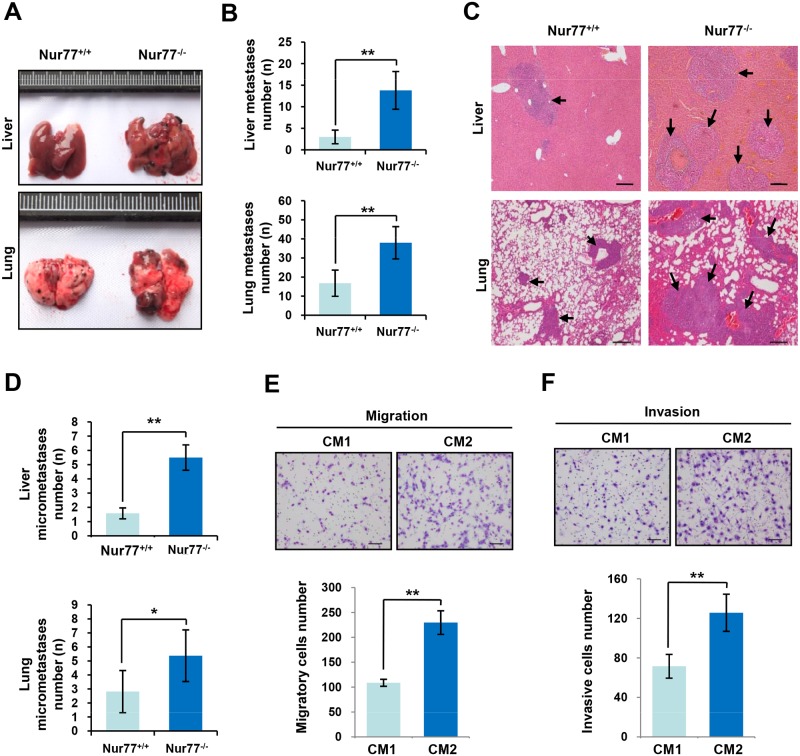
Nur77 deficiency promotes metastasis of B16 melanoma *in vivo* and enhanced migration and invasion of tumor cells *in vitro*. **(A and B)** Representative images (A) and quantitative analysis (B) of liver and lung metastases of B16 melanoma in Nur77^+/+^ and Nur77^-/-^ mice. **(C and D)** H&E-stained liver and lung sections (C) and quantification of liver and lung metastatic foci (D) of Nur77^+/+^ mice or Nur77^-/-^ mice (n = 6) after B16 melanoma inoculation (magnification: ×40). **(E and F)** Migration (E) and invasion (F) assays were performed in CT26 mouse colon carcinoma cells treated with conditioned media derived from Nur77^+/+^ peritoneal macrophages (CM1) or conditioned media derived from Nur77^-/-^ peritoneal macrophages (CM2). Representative images are presented (E and F, top; magnification: ×100) and the relative number of migratory cells (E, bottom) and invasive cells (F, bottom) were counted. Statistical significance was determined by a two-tailed, unpaired Student's *t* test. *p < 0.05, **p < 0.01.

We recently reported that lack of Nur77 increased inflammatory response and contributed to the development of inflammatory diseases including IBD and sepsis [19–21]. Given that aberrant inflammation is implicated in cancer development, we thus hypothesized that host Nur77 deletion might facilitate tumor progression. To test this hypothesis, we injected B16 mouse melanoma cells into the lateral vein in the Nur77^+/+^ and Nur77^-/-^ mice. We found that host genetic deletion of Nur77 in mouse markedly promoted tumor metastasis as revealed by increased macroscopically-visible metastases in the livers and lungs of the Nur77^-/-^ mice 4 weeks after inoculation of B16 cells (Fig 1A and 1B) and developed more liver and lung micrometastases (Fig 1C and 1D) compared with Nur77^+/+^ mice. In a subcutaneous xenograft model, the increased spontaneous metastases to the liver and lung were observed in Nur77^-/-^ mice after subcutaneous injection of B16 mouse melanoma cells (S1 Fig). Collectively, these results indicated the lack of Nur77 in mice accelerated tumor metastasis.

### CM from Nur77^-/-^ peritoneal macrophages promotes tumor cells migration and invasion

We have previously demonstrated that Nur77 deficiency in mice leads to systemic inflammation [19]. Therefore, we posited that host Nur77 deletion could promote tumor metastasis by Nur77-mediated inflammation. Interestingly, starting after 24 h of treatment with conditioned media (CM) derived from peritoneal macrophages of Nur77^+/+^ and Nur77^-/-^ mice, we observed that the conditioned media derived from Nur77^-/-^ peritoneal macrophages (CM2) significantly enhanced the migratory ability of CT26 mouse colon carcinoma cells compared with the conditioned media derived from Nur77^+/+^ peritoneal macrophages (CM1) (Fig 1E). In agreement with this result, invasion assay revealed that CM2 potently enhanced CT26 cells invasive property (Fig 1F). These results suggested that inflammation elicited by lack of Nur77 likely contributed to tumor cells migration, invasion and metastasis.

### TNF-α is required for CM2-mediated EMT of tumor cells

**Fig 2:**
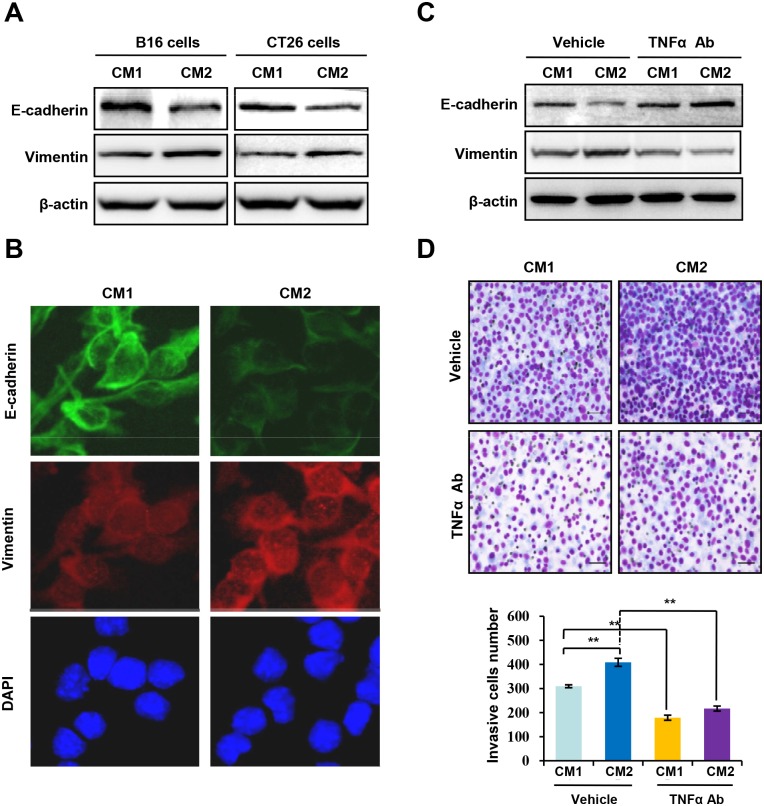
CM2-induced EMT of tumor cells is dependent on inflammatory cytokine TNF-α. **(A)** Western blotting of E-cadherin, Vimentin, and β-actin in B16 and CT26 cells treated with conditioned media derived from Nur77^+/+^ peritoneal macrophages (CM1) or conditioned media derived from Nur77^-/-^ peritoneal macrophages (CM2). **(B)** Immunofluorescent staining of E-cadherin (green) and Vimentin (red) in CT26 cells treated with CM1 or CM2. Nuclei were stained with DAPI (blue). Representative images are presented (magnification: ×400). **(C)** Western blotting of E-cadherin, Vimentin, and β-actin in CT26 cells treated with CM1 or CM2 in the absence or presence of TNF-α antibody. **(D)** An invasion assay was performed in CT26 cells treated with CM1 or CM2 in the absence or presence of TNF-α blocking antibody. Representative images are presented (top; magnification: ×100) and the relative number of invasive cells (bottom) were counted. Statistical significance was determined by a two-tailed, unpaired Student's *t* test. **p < 0.01.

Since host Nur77 deletion is involved in tumor cells metastasis, it is possible that host Nur77 deletion may regulate EMT, which is a key event in tumor metastasis. Thus, we analyzed the effect of conditioned media derived from Nur77^+/+^ and Nur77^-/-^ peritoneal macrophages on the expression of E-cadherin, an epithelial marker, and vimentin, a mesenchymal marker, both of which are important for the EMT process. Our results showed that treatment of B16 and CT26 cells with CM2 significantly reduced E-cadherin expression, but increased Vimentin expression compared with CM1 (Fig 2A), which were further validated by immunofluorescence staining (Fig 2B), Together, these results indicate a crucial role of host Nur77 deletion in regulating the EMT of cancer cells.

Inflammatory cytokines in TME contribute to tumor progression [25]. Interestingly, using an antibody against TNF-α in CM, we found that TNF-α blocking antibody greatly inhibited CM2-induced EMT. As shown in Fig 2C, treatment of CT26 cells with CM2 markedly induced E-cadherin losses and Vimentin increases, which were potently reversed by the TNF-α blocking antibody, thus suggesting that the inflammatory cytokine TNF-α was responsible for CM2-induced EMT. Indeed, the addition of TNF-α to CM1 greatly induced E-cadherin reduction and vimentin upregulation (S2 Fig), which is similar to that of CM2.

We further examined whether inhibition of TNF-α was involved in CM2-driven invasive behavior of tumor cells. Results from invasion assays showed that CM2 significantly enhanced the invasive ability of CT26 cells. However, CM2 showed only a limited effect on CT26 invasion upon treatment with TNF-α blocking antibody (Fig 2D, **top**). These findings were also summarized in Fig 2D, **bottom**. Together, these results suggested that lack of Nur77 in macrophages activated EMT and promoted tumor cells migration and invasion through regulating the secretion of the inflammatory cytokine TNF-α.

### Lack of inflammatory cells infiltrate in tumors from primary B16 melanoma in Nur77^-/-^ mice

**Fig 3:**
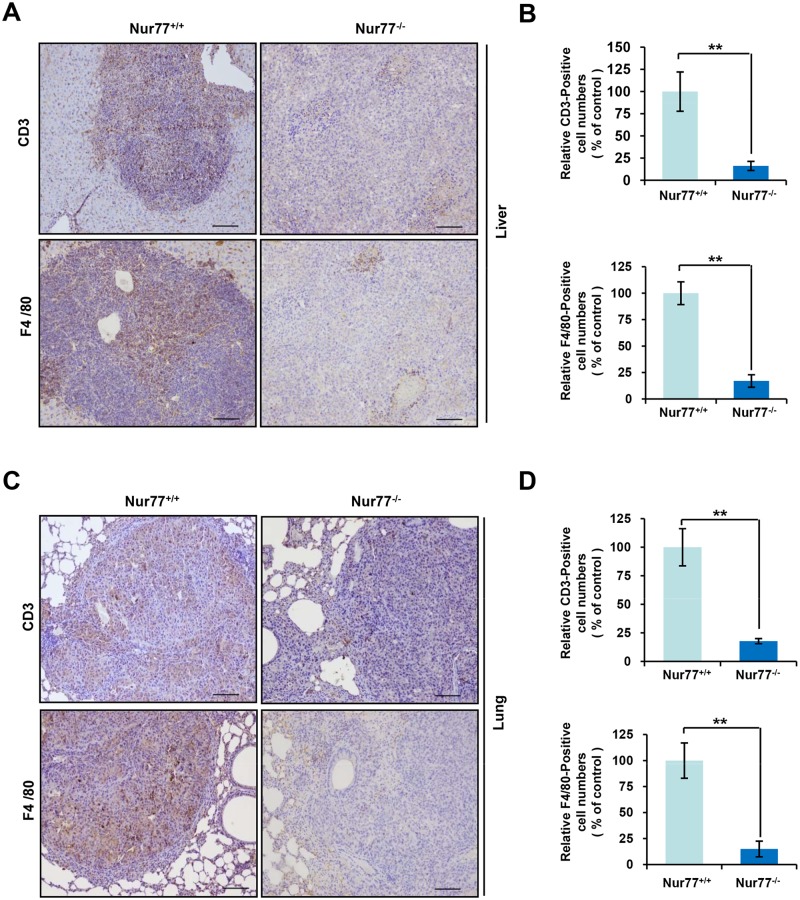
Liver and lung metastases of B16 melanoma from Nur77^-/-^ mice lack T lymphocyte and macrophage infiltration. **(A-D)** Specimens of liver (A and B) and lung (C and D) tissues from Nur77^+/+^ and Nur77^-/-^ mice were stained with anti-CD3 or anti-F4/80 antibody. Representative images are shown (A and C; magnification, ×100), and the relative numbers of CD3-positive or F4/80-positive cells were calculated (B and D). Statistical significance was determined by a two-tailed, unpaired Student's *t* test. **p < 0.01.

Tumor-infiltration by inflammatory cells is a constant feature [1, 4–7]. Unexpectedly, histology of B16 tumors from Nur77^-/-^ mice showed very little inflammatory cells accumulation. As shown Fig 3A and 3B, we stained the B16 tumors from Nur77^+/+^ and Nur77^-/-^ mice with anti-CD3 and anti-F4/80 antibodies to respectively highlight the presence of T lymphocytes and macrophages/DCs, and found that both T lymphocytes and macrophages/DCs showed a massive accumulation in the tumors of liver metastases in Nur77^+/+^ mice, while the percentage of both these inflammatory cells in the tumors of liver metastases was significantly reduced in Nur77^-/-^ mice. Consistent with these results, reduced infiltration by these inflammatory cells was observed in the tumors of lung metastases in Nur77^-/-^ mice compared with Nur77^+/+^ mice (Fig 3C and 3D). In subcutaneous xenograft model, it was also observed that B16 melanoma growing in the Nur77^-/-^ mice lacked T lymphocyte and macrophage infiltration (S3 Fig). Together, these data suggest that absence of inflammatory infiltrate may accelerate tumor metastasis in the Nur77^-/-^ host due to inefficient immune response.

### Macrophages lacking Nur77 have impaired migratory ability

**Fig 4:**
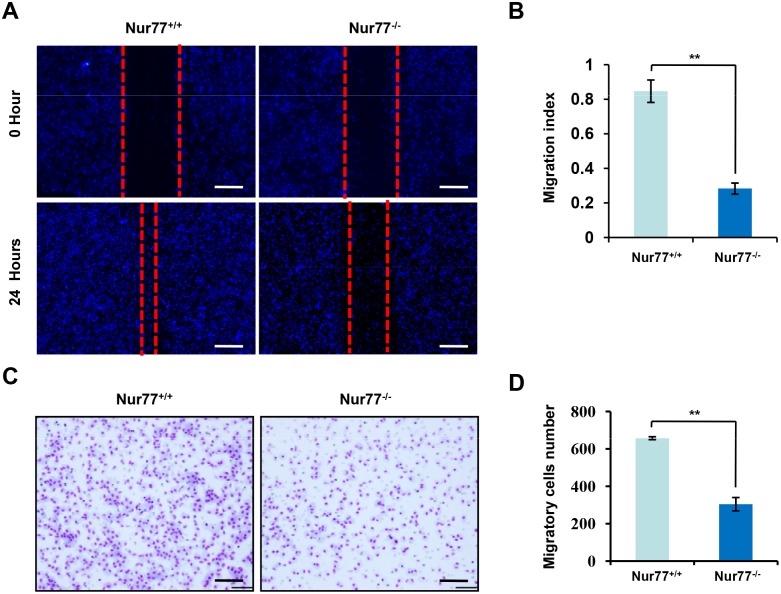
Peritoneal macrophages lacking Nur77 have impaired migratory ability. **(A and B)** Peritoneal macrophages from Nur77^+/+^ or Nur77^-/-^ mice were wounded by a 20 μL plastic pipette tip and cultured in serum-free medium for the indicated time, and cell migration into the wounded area was evaluated (A). Migration index was calculated (B). **(C and D)** For the migration assay, peritoneal macrophages from Nur77^+/+^ or Nur77^-/-^ mice were plated in a chamber for 24 hours, then the migratory cells were stained and observed. Representative images are presented (C; magnification: ×100) and the relative number of migratory cells (D) were counted. Statistical significance was determined by a two-tailed, unpaired Student's *t* test. **p < 0.01.

Given that lack of inflammatory cells infiltration in Nur77^-/-^ mice was so striking, we thus hypothesized that Nur77 deletion might cause a more profound defect in inflammatory cells migration. We first performed an *in vitro* wound healing experiment with peritoneal macrophages from Nur77^+/+^ and Nur77^-/-^ mice. As shown in Fig 4A and 4B, the peritoneal macrophages were layered in a culture dish and, after reaching confluence, the monolayer was wounded with a pipette tip. The peritoneal macrophages from Nur77^+/+^ mice migrated from both sides of the wound and within 24 hours reestablished continuity of the monolayer. However, the peritoneal macrophages from Nur77^-/-^ mice were unable to repopulate the wound within 24 hours, which was consistent with the observations made in the migration assay (Fig 4C and 4D). Conversely, overexpression of Nur77 significantly enhanced macrophage migration (S4 Fig). Collectively, these results suggested that lack of Nur77 significantly reduced the ability of macrophages migration.

### CSF-1R is a novel target gene of Nur77 in monocytes and macrophages

**Fig 5:**
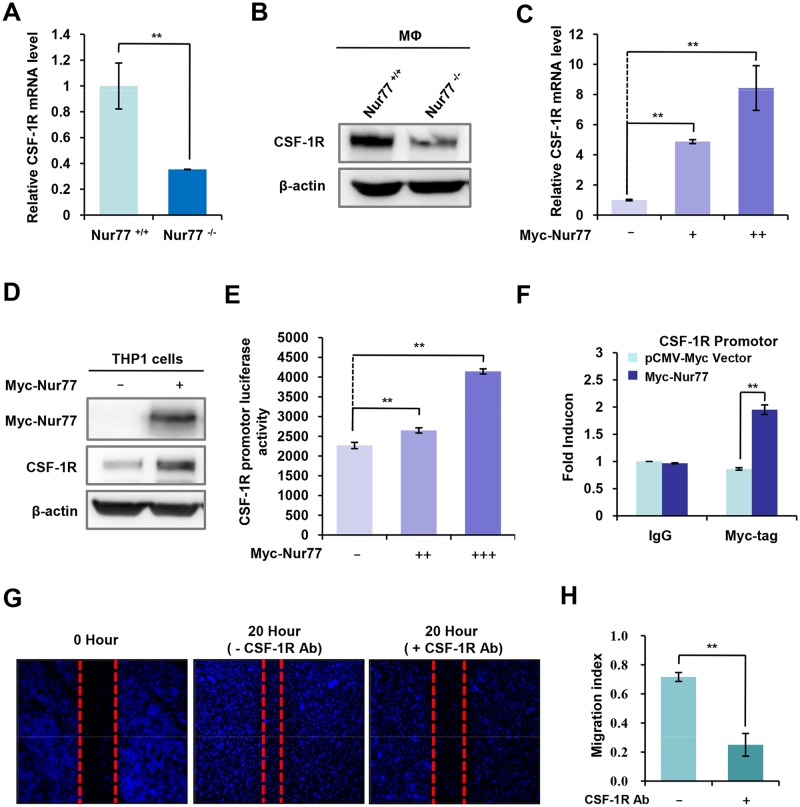
Nur77 transcriptionally regulates CSF-1R expression in monocytes and macrophages. **(A and B)** The mRNA (A) and protein (B) levels of CSF-1R were detected in peritoneal macrophages from Nur77^+/+^ or Nur77^-/-^ mice. **(C and D)** The mRNA (C) and protein (D) levels of CSF-1R were examined in Nur77-overexpressed THP-1 cells. **(E)** The luciferase activity of CSF-1R promoter was measured in HEK293T cells transfected with the increasing levels of Nur77. **(F)** Binding of Nur77 to the CSF-1R promoter by chromatin immunoprecipitation (ChIP) assay. ChIP assays were performed using anti-Myc tag antibody in the THP-1 cells transfected Myc-Nur77 or vector, and then qPCR with primers specifically targeting CSF-1R promoter regions. ChIP signals were presented as fold enrichment over that of IgG. **(G and H)** Peritoneal macrophages from Nur77^+/+^ mice were wounded by a 20 μL plastic pipette tip, and then treated with CSF-1R-specific antibody for the indicated time or left untreated. Cell migration into the wounded area was evaluated (G). Migration index was calculated (H). Statistical significance was determined by a two-tailed, unpaired Student's *t* test. **p < 0.01.

It has been shown that the colony-stimulating factor-1 (CSF-1) and its receptor CSF-1R sustain monocytes/macrophages survival, proliferation, and motility, and also contribute to tumor progression [26, 27]. Thus, we hypothesized that Nur77 might affect the migratory ability of macrophages by regulating CSF-1R expression. Interestingly, we found that the mRNA (Fig 5A) and protein (Fig 5B) levels of CSF-1R were significantly decreased in peritoneal macrophages from Nur77^-/-^ mice as compared to peritoneal macrophages from Nur77^+/+^ mice. On the contrary, overexpression of Nur77 in RAW264.7 mouse macrophages greatly enhanced CSF-1R mRNA (Fig 5C) and protein (Fig 5D) levels in a dose-dependent way. Moreover, we observed the luciferase activity of CSF-1R promotor was dose-dependently enhanced by Nur77 (Fig 5E). These results indicated that Nur77 induced CSF-1R expression at the transcriptional level. Because Nur77 often functions as a transcriptional factor to bind its target genes' promoter regions and regulate their expression [15, 28], we speculated that Nur77 might bind CSF-1R promoter region to modulate its expression. To test this hypothesis, we performed chromatin immunoprecipitation assays to analyze the ability of Nur77 protein to bind to CSF-1R promoter. Expectably, our results showed that Nur77 binding increased significantly on the CSF-1R promotor region in Nur77-overexpresssed cells compared with vector-overexpressed control cells (Fig 5F). Next, it was determine whether CSF-1R is involved in macrophage migration. Interestingly, blockage of CSF-1R function using CSF-1R-specific antibody significantly impaired the migratory ability of macrophages (Fig 5G and 5H), indicating that CSF-1R mediates macrophage migration. Taken together, our results indicated that CSF-1R is a target gene of Nur77, and may contribute to Nur77-mediated motility of macrophages.

## Discussion

Tumor metastasis is the major cause of human cancer death, and involves the detachment and exudation of cancer cells from the primary tumor, dissemination to distant organ sites and adaptation to foreign environments [29]. Each of these processes is dictated by the cooperation between tumors and their microenvironment. Although various inflammatory and immune cells are enriched in the TME, they fail to exercise anti-tumor effector functions, and thus promote tumor growth and metastasis [1, 2, 30]. The underlying mechanisms of the inflammatory and immune cells in TME that drive tumor development and progression are important issues that need to be further examined. In this study, we investigated the role of host Nur77 in antitumor response. Our results revealed that the expression of host Nur77 is required for its antitumor response. Host genetic deletion of Nur77 in mice significantly enhanced B16 melanoma cells metastasis to the lung and liver, which was consistent with a recent report that Nur77-deficient mice that specifically lacked "patrolling" monocytes" (PMo) showed increased cancer lung metastasis *in vivo* [31].

A major reason for the ability of Nur77^-/-^ hosts to enhance tumor metastasis was likely the increased pro-inflammatory response. We recently demonstrated that Nur77 deficiency in elderly mice resulted in systemic inflammation [19], and lack of Nur77 in mice was associated with increased risk for inflammatory diseases including IBD and sepsis [20, 21]. Here, we further revealed that inflammation caused by Nur77 deficiency in mice contributes to tumor metastasis. TNF-α, a main pro-inflammatory cytokine in the TME, has been proposed to enhance tumor development and progression [8, 9]. One important finding here is that TNF-α was indispensable for cancer cells migration and invasion by Nur77-elicited inflammation. Conditioned media derived from Nur77^-/-^ peritoneal macrophage (CM2) significantly promotes cancer cells migration and invasion compared with conditioned media derived from Nur77^+/+^ peritoneal macrophage (CM1), which is greatly reversed by TNF-α blocking antibody. Wu *et al* recently reported that the inflammatory TME contributes to breast cancer metastasis through a novel mechanism wherein TNF-α regulates Snail stabilization through the activation of NF-κB pathway [32]. Here, we revealed that CM2 promotes cancer cells to undergo EMT, a crucial event in tumor metastasis. Blocking TNF-α abolished the activity of CM2 in inducing EMT of cancer cells, strengthening the notion that the inflammatory TME plays an important role not only in tumorigenesis, but also in tumor metastasis.

Infiltration by inflammatory and immune cells is a hallmark of malignant tumors that can play a critical role in mounting an antitumor response [33]. Surprisingly, we observed that liver and lung metastases of B16 melanoma in Nur77^-/-^ mice showed very little inflammatory infiltrate. On the contrary, livers and lungs from Nur77^+/+^ mice showed diffuse signs of inflammation. These findings suggest that an inefficient antitumor response due to absence of inflammatory infiltrate might be another driver of tumor metastasis in Nur77-deficient mice. In addition, Nur77-deficient macrophages showed a reduced ability to migrate, whereas overexpression of Nur77 greatly enhanced the migratory ability of macrophages in *in vitro* wound healing and migration assays, indicating that lack of Nur77 may hinder cell chemotaxis, and thus impairs tumor infiltration. It has recently also been reported that overexpression of Nur77 promotes colorectal cancer cell invasion and metastasis through regulation of MMP-9-dependent E-cadherin reduction. Thus, macrophage migration may share common features with cancer cell invasion and metastasis when Nur77 is overexpressed.

Accumulating evidence suggests that CSF-1 and its receptor, CSF-1R, are implicated in tumor development and progression [26, 27]. CSF-1 binds to CSF-1R and sustains inflammatory cell proliferation and motility in TME. Blocking CSF-1 expression in a xenograft model significantly decreased the growth capacity of tumor cells [34]. CSF-1R inhibition, either pharmacologic with CSF-1R-specific inhibitor or genetic downregulation with specific siRNA, blocked migration of macrophage induced by prostaglandin (PGE2) [35], a key mediator of immunity and inflammation. Interestingly, we here further uncovered that CSF-1R was a novel target gene of Nur77. Using ChIP assays, we determined that Nur77 bound to CSF-1R promoter to enhance its expression in macrophages and monocytes. Notably, Nur77-deficient macrophages or monocytes exhibited low levels of CSF-1R expression. CSF-1R plays an important role in regulating migration of inflammatory and immune cells such as macrophages and lymphocytes [35, 36]. The present results showed that infiltration of macrophages/DCs and T lymphocytes in the tumors of Nur77^-/-^ mice was markedly abrogated relative to Nur77^+/+^ mice. *In vitro* studies further reveal that blockage of CSF-1R function using CSF-1R-specific antibody greatly impaired the migratory ability of inflammatory cells. It is here speculated that the low expression of CSF-1R in inflammatory and immune cells likely attenuated their migratory ability and ability to infiltrate into tumor, and thus facilitate tumor metastasis in Nur77^-/-^ mice.

In summary, we have identified Nur77 as a non-redundant host factor in *in vivo* anticancer response. Our study provides novel mechanisms through which inflammatory cells promote tumor cells invasion and metastasis. On one hand, Nur77-deficient inflammatory cells secrete TNF-α to drive tumor cells to undergo EMT. On the other hand, lack of Nur77 results in low levels of CSF-1R expression, which reduces the migratory capacity of inflammatory cells and subsequently hinders cell chemotaxis and tumor infiltration. These findings thus highlight the complex role of inflammation in host-tumor interaction.

## Supporting information

S1 Fig Lack of Nur77 in mice promotes metastasis of tumor cells after subcutaneous injection of B16 mouse melanoma cells. H&E-stained liver and lung sections and representative images were shown (magnification: ×40).
(TIF)

S2 Fig TNF-α induces EMT of tumor cells. Western blotting of E-cadherin, vimentin, and β-actin in B16 and CT26 cells treated with conditioned media derived from Nur77^+/+^ peritoneal macrophages (CM1) in presence or absence of TNF-α (50 ng/μL) for 24 h.
(TIF)

S3 Fig B16 melanoma growing in the Nur77^-/-^ mice lack T lymphocyte and macrophage infiltrate. The tumor tissues derived from B16 melanoma cells were stained with anti-CD3 or anti-F4/80 antibody. Representative images are shown (magnification, ×200).
(TIF)

S4 Fig Overexpression of Nur77 promotes macrophage migration. **(A and B)** RAW264.7 cells were transfected with vector or Nur77 plasmid and then wounded using a 20 μL plastic pipette tip and cultured in serum-free medium for the indicated time, and cell migration into the wounded area was evaluated (A). The migration index was calculated (B). **(C and D)** For the migration assay, RAW264.7 cells were transfected with vector or Nur77 plasmid and were then were plated in a chamber for 24 h. Then the migratory cells were stained and observed. Representative images are presented (C; magnification: ×100) and the relative number of migratory cells was determined (D). Statistical significance was determined using a two-tailed, unpaired Student's *t* test. **P < 0.01.
(TIF)
