# Correction: Nur77 deficiency in mice accelerates tumor invasion and metastasis by facilitating TNFα secretion and lowering CSF-1R expression

**DOI:** 10.1371/journal.pone.0173838

**Published:** 2017-03-07

**Authors:** Xiu-Ming Li, Jing-Ru Wang, Tong Shen, Shang-Shang Gao, Xiao-Shun He, Jiang-Nan Li, Tian-Yu Yang, Shen Zhang, Wen-Juan Gan, Jian-Ming Li, Hua Wu

The second affiliation for the tenth author is not indicated. Jian-Ming Li is also affiliated with: Pathology Center and Department of Pathology, Soochow University, Suzhou, China.

## References

[1] Li X-M, Wang J-R, Shen T, Gao S-S, He X-S, Li J-N, et al (2017) Nur77 deficiency in mice accelerates tumor invasion and metastasis by facilitating TNFα secretion and lowering CSF-1R expression. PLoS ONE 12(2): e0171347 doi: 10.1371/journal.pone.0171347 28170411 10.1371/journal.pone.0171347 PMC5295676

