# Supplementary figures and images for: Nur77 deficiency in mice accelerates tumor invasion and metastasis by facilitating TNFα secretion and lowering CSF-1R expression
Source: PLoS One. 2017 Feb 7;12(2):e0171347. doi: 10.1371/journal.pone.0171347 (PMC5295676; doi:10.1371/journal.pone.0171347)

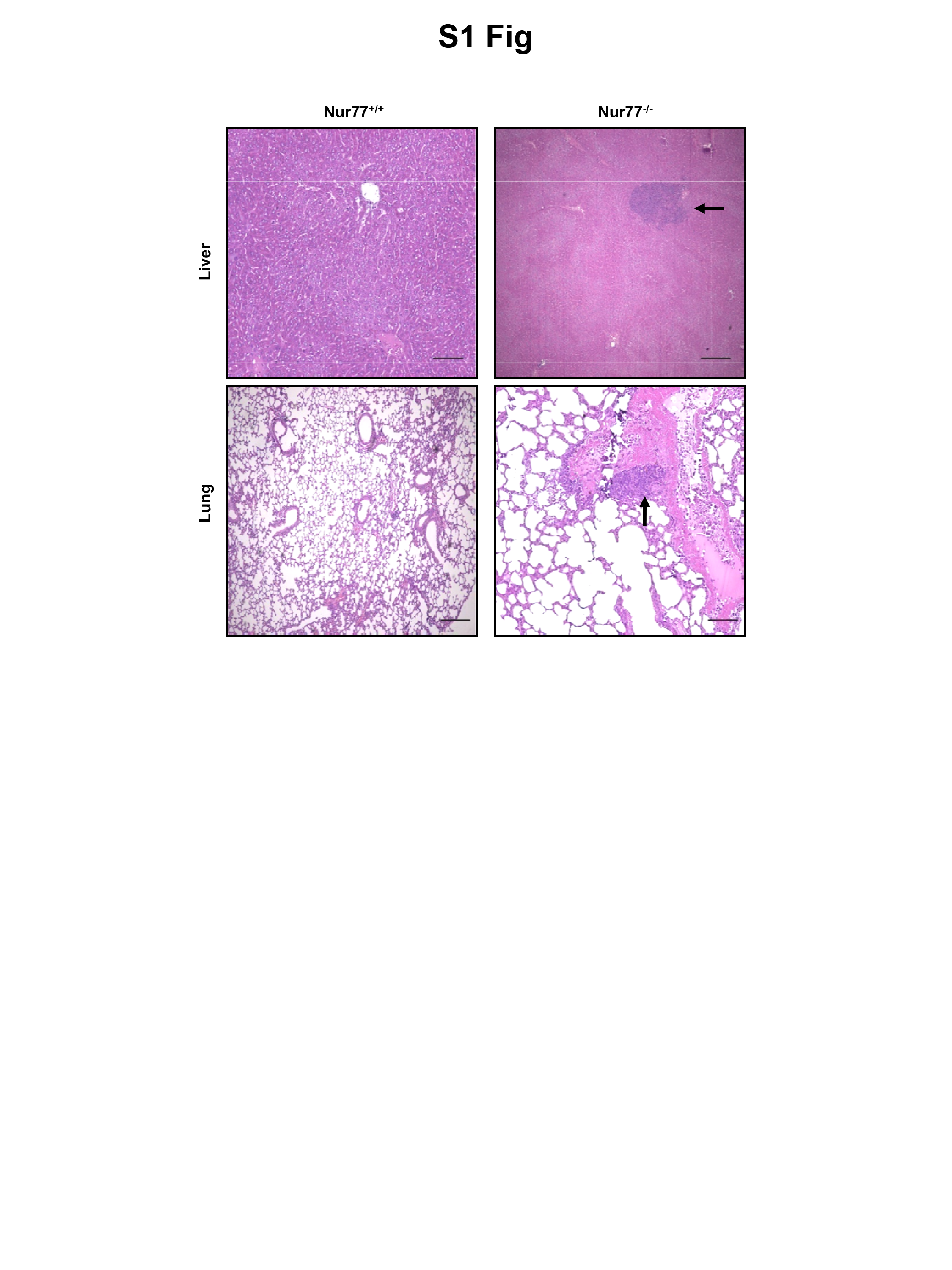

Supplement: S1 Fig — H&E-stained liver and lung sections and representative images were shown (magnification: ×40). (TIF) [file pone.0171347.s001.tif]

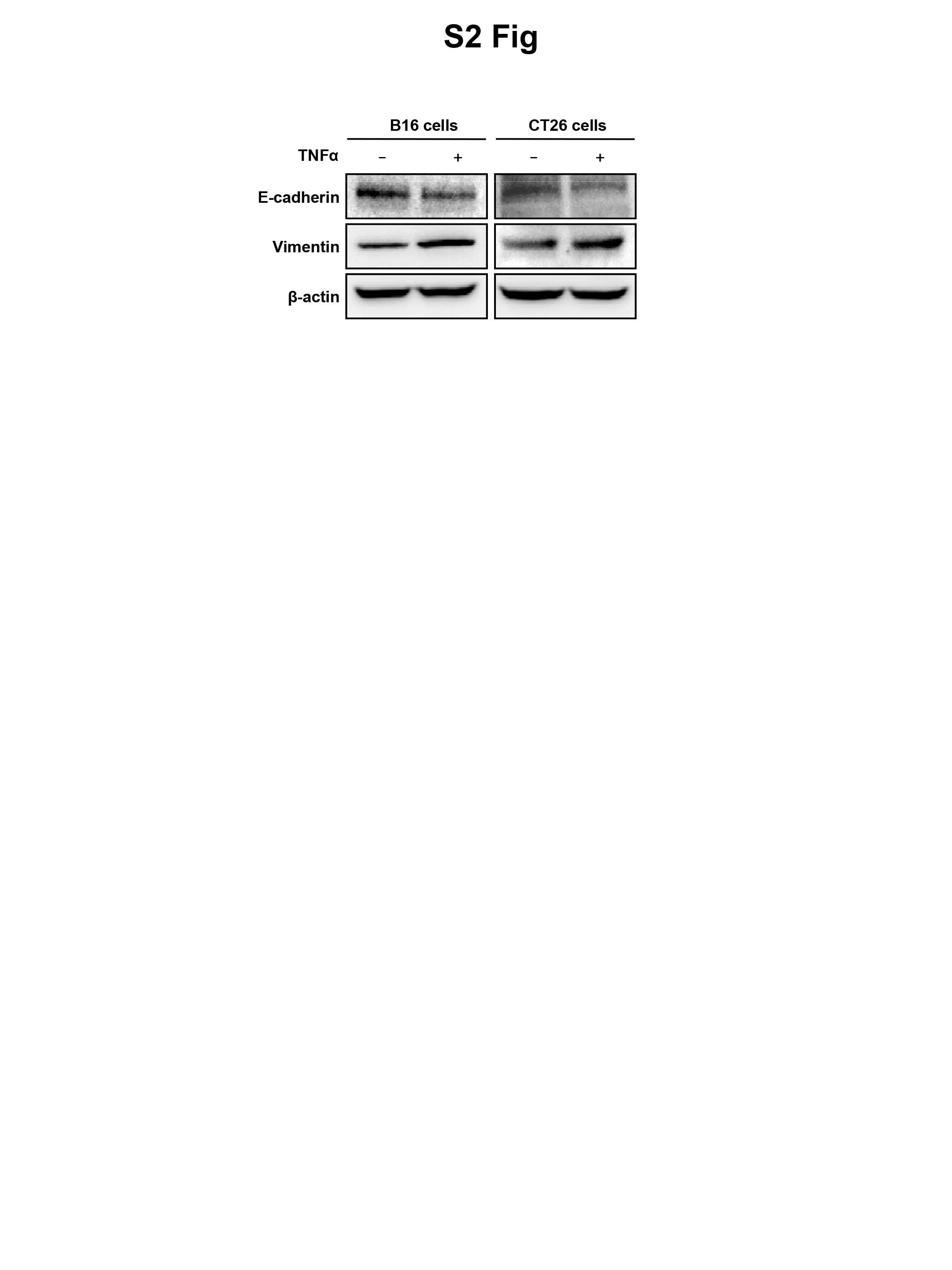

Supplement: S2 Fig — Western blotting of E-cadherin, vimentin, and β-actin in B16 and CT26 cells treated with conditioned media derived from Nur77+/+ peritoneal macrophages (CM1) in presence or absence of TNF-α (50 ng/μL) for 24 h. (TIF) [file pone.0171347.s002.tif]

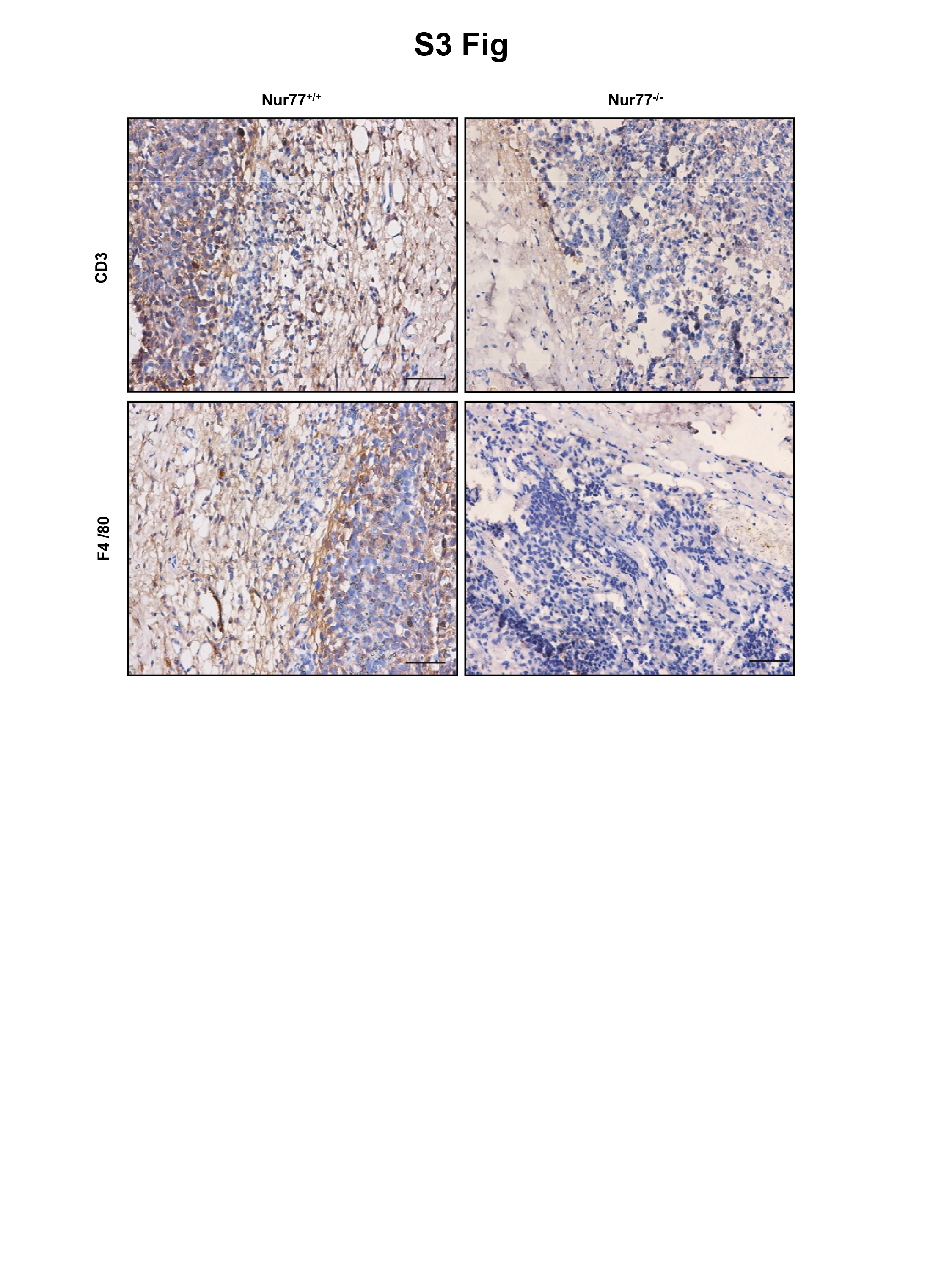

Supplement: S3 Fig — The tumor tissues derived from B16 melanoma cells were stained with anti-CD3 or anti-F4/80 antibody. Representative images are shown (magnification, ×200). (TIF) [file pone.0171347.s003.tif]

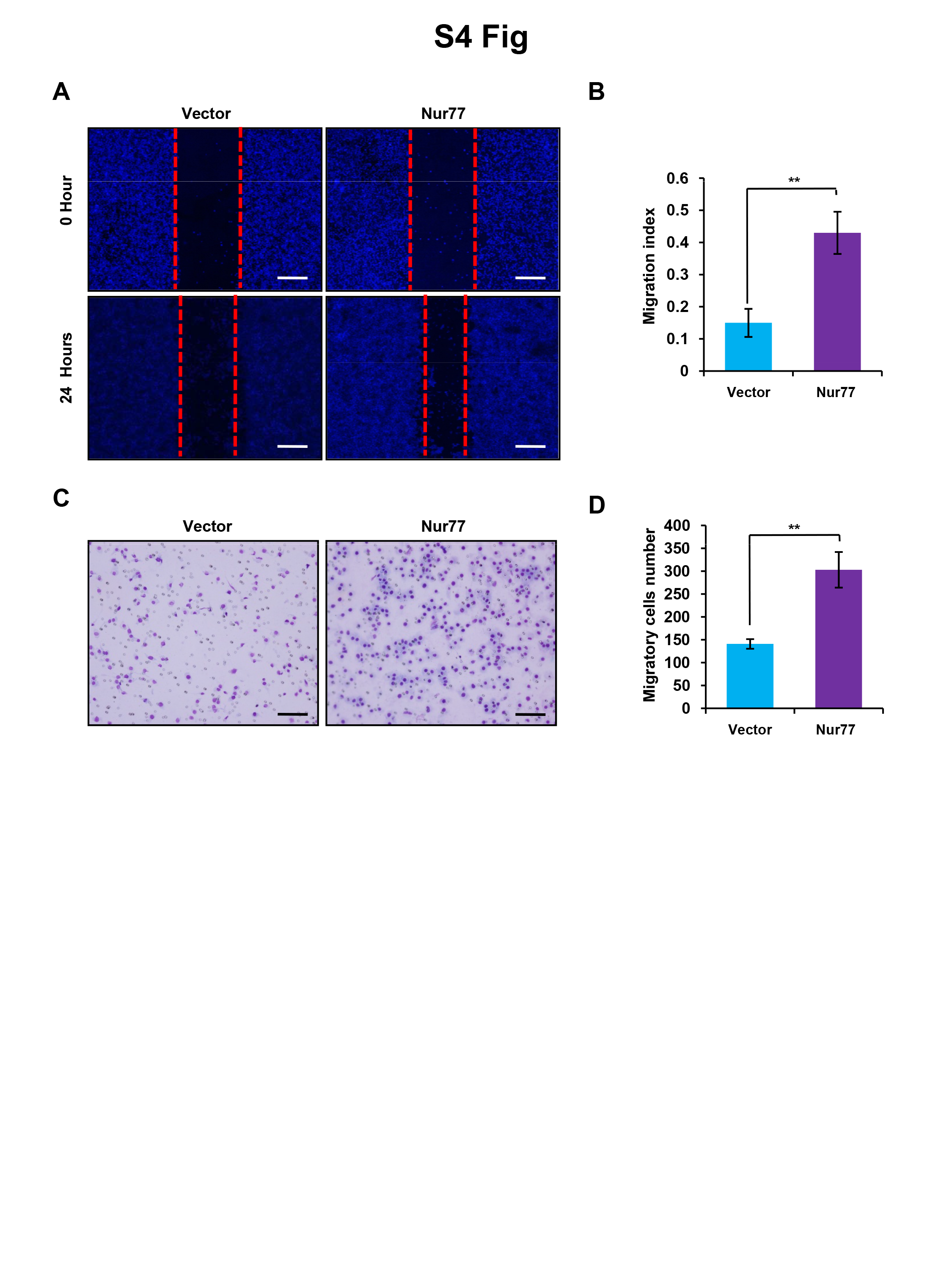

Supplement: S4 Fig — (A and B) RAW264.7 cells were transfected with vector or Nur77 plasmid and then wounded using a 20 μL plastic pipette tip and cultured in serum-free medium for the indicated time, and cell migration into the wounded area was evaluated (A). The migration index was calculated (B). (C and D) For the migration assay, RAW264.7 cells were transfected with vector or Nur77 plasmid and were then were plated in a chamber for 24 h. Then the migratory cells were stained and observed. Representative images are presented (C; magnification: ×100) and the relative number of migratory cells was determined (D). Statistical significance was determined using a two-tailed, unpaired Student's t test. **P < 0.01. (TIF) [file pone.0171347.s004.tif]
